# Recent Advances of CRISPR/Cas9-Based Genetic Engineering and Transcriptional Regulation in Industrial Biology

**DOI:** 10.3389/fbioe.2019.00459

**Published:** 2020-01-28

**Authors:** Shangjie Zhang, Feng Guo, Wei Yan, Zhongxue Dai, Weiliang Dong, Jie Zhou, Wenming Zhang, Fengxue Xin, Min Jiang

**Affiliations:** ^1^State Key Laboratory of Materials-Oriented Chemical Engineering, College of Biotechnology and Pharmaceutical Engineering, Nanjing Tech University, Nanjing, China; ^2^Jiangsu National Synergetic Innovation Center for Advanced Materials (SICAM), Nanjing Tech University, Nanjing, China

**Keywords:** industrial biology, CRISPR/Cas9, synthetic biology, gene circuits, genetic engineering

## Abstract

Industrial biology plays a crucial role in the fields of medicine, health, food, energy, and so on. However, the lack of efficient genetic engineering tools has restricted the rapid development of industrial biology. Recently, the emergence of clustered regularly interspaced short palindromic repeats/CRISPR-associated protein 9 (CRISPR/Cas9) system brought a breakthrough in genome editing technologies due to its high orthogonality, versatility, and efficiency. In this review, we summarized the barriers of CRISPR/Cas9 and corresponding solutions for efficient genetic engineering in industrial microorganisms. In addition, the advances of industrial biology employing the CRISPR/Cas9 system were compared in terms of its application in bacteria, yeast, and filamentous fungi. Furthermore, the cooperation between CRISPR/Cas9 and synthetic biology was discussed to help build complex and programmable gene circuits, which can be used in industrial biotechnology.

## Introduction

**Figure 1:**
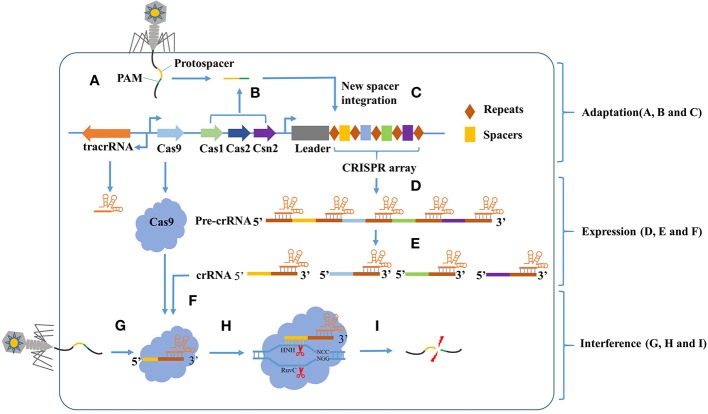
The CRISPR-Cas9 mediated adaptive immunity is divided into three phases: Adaptation, Expression, and Interference. In the adaptation phase, **(A)** the host is invaded by phage DNA during infection. **(B)** Subsequently, the invading DNA is processed by various Cas genes into small DNA fragments (protospacer). The selection of protospacer depends in part on the specific recognition of protospacer adjacent motif (PAM) present within the viral genome. **(C)** The small DNA fragments is then incorporated into the CRISPR locus of the bacterial genome as a new spacer, flanked by a repeat sequence. In the expression phase, **(D)** the CRISPR locus is transcribed into a long precursor CRISPR RNA (pre-crRNA). **(E)** Then, the tracrRNA combine to pre-crRNA to a dual RNA hybrid, this dual RNA hybrid will be recognized and cut by RNase III with the existence of Cas9 protein, resulting in mature crRNA. **(F)** The mature crRNA will combine to Cas9 protein and guide the DNA cleavage. In the Interference phase, when phage DNA invades again, the Cas9:crRNA complex identifies a sequence that is complementary to the spacer and adjacent to the PAM. **(I)** Finally, the invading DNA is cleaved by Cas9 protein to prevent infection.

Industrial biotechnology has advanced significantly in recent years due to the improvement of genomic engineering tools. Genetic engineering is a complex technology that manipulates genes at molecular level. The recombined exogenous genes can be replicated, transcribed, translated, and expressed in receptor cells to produce various valuable chemicals. During the long-time evolution of microorganisms, a unique adaptive immune system, named as clustered regularly interspaced short palindromic repeat sequences and CRISPR-associated protein 9 (CRISPR/Cas9), was employed by bacteria and archaea to defend against foreign-invading DNA (Figure 1; Horvath and Barrangou, 2010). This system is consisted of a Cas9 nuclease, a target-recognizing CRISPR RNA (crRNA), and auxiliary non-coding trans-activating crRNAs (tracrRNAs) (Jiang and Doudna, 2017). The precursor crRNA (pre-crRNA) is able to combine with several tracrRNAs, and can be recognized and processed by RNase III into mature crRNA::tracrRNAs (dual RNA hybrid). A single-guide RNA (sgRNA) can be constructed by fusing a crRNA containing the targeting guide sequence to a tracrRNA and then combines to Cas9 protein, generate DNA double-strand breaks (DSBs), and then alter the target gene by cellular DNA repair mechanisms (Cong et al., 2013). The DSBs can be repaired through two different ways: non-homologous end-joining (NHEJ) and homologous repair (HR) (Capecchi, 1989). NHEJ can introduce insertion and deletion at the target site. HR uses a foreign DNA donor template to recombine with the target site for introducing a specific point mutation or insertion of the desired sequence.

Previous methods of manufacturing targeted DSBs relied on protein–DNA recognition systems, such as zinc-finger nucleases (ZFNs) and transcription activator-like effector nucleases (TALENs). However, these systems were limited due to their complex and expensive operation. On the contrary, the CRISPR/Cas9 system has been widely employed in various fields owing to its high efficiency, low cost, and convenience (Gaj et al., 2013; Hsu et al., 2014). Hence, in this review, the potential of CRISPR/Cas9 in industrial biotechnology was demonstrated by introducing its applications in bacteria, yeasts, and filamentous fungi. The future prospect of cooperation between CRISPR/Cas9 and synthetic biology to build complex and programmable gene circuits was also summarized.

## Versatile Designs of CRISPR/Cas9 for Highly Efficient Gene Editing

Although the CRISPR/Cas9 system has been successfully used in bacteria, yeasts, and fungi, its gene editing efficiency is still unsatisfactory. How to improve the gene editing efficiency has been the focus in this filed. Several strategies employed to improve genome editing efficiency with CRISPR/Cas9 were accordingly summarized.

### Improvement of Homologous Recombination Efficiency

The mechanism of gene editing is repairing the DSBs generated by CRISPR/Cas9 through NHEJ or HR. Once DSBs occurs, most industrial microorganisms prefer the NHEJ pathway over HR even with exogenous donors, which retards the precise genome editing. In order to increase the frequency of HR, two main strategies were employed: (1) coupling the CRISPR/Cas9 system to lambda Red oligonucleotide recombineering and (2) deleting *KU70* or *KU80* heterodimer involved in NHEJ repair. For instance, Jiang et al. established a two-plasmid-based CRISPR/Cas9 system in *Escherichia coli*, in which *Streptococcus pyogenes* Cas9 and crRNA array were expressed in the low-copy plasmid (pCas) and high-copy plasmid (pCRISPR) series (Jiang et al., 2013; Mali et al., 2013). Although this novel genetic engineering tool had a better performance than did the traditional one, it still needed further modifications to obtain higher efficiency. Based on this system, a triple-plasmid strategy was designed with the third plasmid carrying the λ-Red genes expressed from ParaB. In contrast, this three-plasmid system increased the percentage of mutant cells from 19 to 65%. In another study, a CRISPR/Cas9 system, which had 94% efficiency toward single-gene non-sense mutations, was accordingly established in *Komagataella pastoris*. However, the integration efficiency was really low (2%), when a donor template with 1-kbp homologous arms was provided (Weninger et al., 2016). To improve the integration efficiency with markerless donor cassettes, the *KU70* gene was accordingly knocked out and improved the knock-in efficiency up to nearly 100% (Weninger et al., 2018).

### Adoption of Optimal Promoter for Expression of Cas9 and gRNA

**Figure 2:**
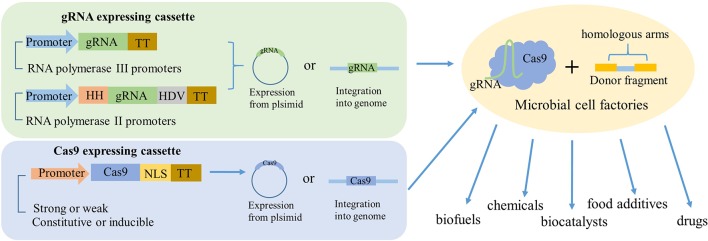
Schematic to illustrate how gRNA and Cas9 expressed in industrial microorganisms. The gRNA was mainly expressed by two ways. One is directly derived by RNA polymerase III promoters. Another one is flanking the gRNA with HH and HDV ribozymes and then derived by RNA polymerase II promoters. The Cas9 could be expressed by a strong or weak, constitutive, or inducible promoter and fused with nuclear localization sequence (NLS). The two expressing cassettes can be expressed either from the same plasmid, separate plasmids, or integrated into the genome, most time with a donor fragment. So, after the genetic engineering, the industrial microorganisms could be transformed to high-powered microbial cell factories to product valuable production, such as biofuels, chemicals, biocatalysts, food additives, drugs, and so on.

In most studies, Cas9 protein and gRNA were separated into independent vectors. The Cas9 protein was commonly expressed in a low-copy plasmid with constitutive promoters, because high-level expression of Cas9 will lead to negative influence on microbial growth. In contrast, the expression of gRNA should choose high-copy plasmids with a strong promoter. The RNA polymerase III (pol III) promoters had been successfully employed in many cases; however, it was difficult to find suitable RNA pol III promoters. Thus, the sgRNA was flanked with two ribozyme sequences, 5′ end hammerhead (HH) and 3′ end hepatitis delta virus (HDV) to express under a strong RNA polymerase II promoter (Figure 2; Nødvig et al., 2015). In addition, synthesized hybrid promoters provide another feasible substitute for gRNA expression (Cai et al., 2019). For instance, the gene editing efficiency by harnessing the common RNA pol III promoter SNR52 to express sgRNA in oleaginous yeast *Yarrowia lipolytica* only reached 26%. In order to optimize the expression of sgRNA, Schwartz et al. constructed an RNA polymerase II (Pol II) TEF promoter for sgRNA with HH and HDV ribozymes in 5′ end and 3′ end, and fused the Pol III promoters RPR1, SCR1, and SNR52 with a glycine tRNA (tRNA^Gly^) (Schwartz et al., 2015). Finally, the highest disruption efficiency of 92% reached with synthetic SCR1′-tRNA^Gly^ promoter. In addition, the disruption efficiency using the SNR52′-tRNA^Gly^ promoter was improved by 28% than the initial SNR52 promoter.

### Other Methods

#### Optimization of Cas9 Protein Codons

The importance of codon optimization of Cas9 is different in different strains. For example, the natural *S. pyogenes* Cas9 has shown high targeting efficiency in *Saccharomyces cerevisiae* (Bao et al., 2015). However, the targeting efficiency was unsatisfactory when using native *S. pyogenes* Cas9 in *K. pastoris*. By employing the human codon optimized Cas9 (*Hs*Cas9), the targeting efficiency improved from 32 to 73%, indicating that optimization of Cas9 protein codons plays a crucial role in improving the targeting efficiency. Hence, when the performance of the CRISPR/Cas9 system was unsatisfactory, optimization of Cas9 codons may be a good solution.

#### Adoption of Suitable sgRNA Binding Sites

For industrial microorganisms, the sgRNA binding site is crucial in terms of the targeting efficiency. In order to improve the efficiency, different sgRNAs targeting sites should be tested. For instance, Peng et al. found that the targeting efficiency was distributed between 13 and 100% when using six different sgRNAs targeting six different sites of *mepA* gene, and the sgRNAs with the GC content under 60% had better performance. This result showed that different sgRNAs had great influence on editing efficiency and adoption of suitable sgRNA is important for a high success rate. A series of website tools had been set for sgRNA design and summarized in previous literature (Chuai et al., 2017).

#### Prolongation of Incubation

The targeted chromosomal region could escape sgRNA/Cas9 endonuclease activity in some cases. The effective solution is to prolong the incubation under the appropriate selection pressure to generate iterative DSBs until mutagenic repair occurs. To confirm the effect of prolonged incubation on the mutation efficiency, So et al. introduced two plasmids that contained same sgRNA sequence and different length of homologous arms (30 and 500 bp) to the targeted gene into *Bacillus subtilis*, respectively (So et al., 2017). The mutation efficiencies were improved from 85% to almost 100% after incubation for 9 h. Results demonstrated that the mutation efficiency can be maximized by prolonging incubation.

## CRISPR/Cas9-Mediated Genetic Engineering in Industrial Biology

Industrial microorganisms can produce various value-added chemicals from low-cost feedstock including renewable biomass and organic wastes (Yao et al., 2018). To enhance the performances of industrial microorganisms, genetic modifications were usually implemented to construct desired microbial cell factories. Although various methods are available for genetic modifications, it is time-consuming and labor-intensive. Fortunately, as a surprising “gift,” the CRISPR/Cas9 system greatly improved the efficiency of genetic engineering. Here, we reviewed the applications of the CRISPR/Cas9 system in bacteria, yeasts, and filamentous fungi with special emphasis on *E. coli* and *S. cerevisiae*, which were the most commonly used cell factory.

### The Application of CRISPR/Cas9 in Bacteria

*Escherichia coli* is often used to produce a variety of valuable chemicals, drugs, and biofuels in industrial biotechnology. A traditional method of gene knockout in *E. coli* was to adopt the Red homologous recombination system to mediate the homologous recombination of DNA. However, it is inefficient and especially not suitable for recombination of multiple sites (Murphy and Campellone, 2003). To improve the genetic engineering efficiency, Jiang et al. construct a triple-plasmid system as mentioned above. This novel genetic engineering tool significantly improved the efficiency of genetic modification and thus accelerated the development of industrial biology. In previous studies, Cas9 and gRNA were expressed in two plasmids, respectively, as the simultaneous expression would burden the organism metabolism and cause cell death. Hence, Cas9 or gRNA should be repressed before a genome editing event. Cas9 and gRNA can be assembled into one plasmid containing a pBAD promoter, which is repressed by glucose and induced by arabinose and a temperature-sensitive replicon repA101ts, so that transformed *E. coli* could grow on glucose-amended plates and be edited under the induction with 2 g/L of arabinose. This fast and easy procedure for genome editing could perform continuously, as multiple loci only required one plasmid construction and one step of transformation. To further improve the simultaneous editing efficiency of multiple loci, a CRISPR/Cas9-assisted multiplex genome editing technique was developed. The CRISPR/Cas9-assisted multiplex genome editing technique contained three plasmids: pRedCas9 containing both λ-Red recombineering and Cas9 system under the control of pBAD promoter, pMgRNAs containing gRNAs, and pDonorDNAs carrying multiple donor DNA cassettes (Feng et al., 2018). In another versatile study, Li et al. firstly coexpressed a plasmid containing a gRNA targeting the *bla* gene and Cas9 with the λ-Red recombineering system into *E. coli* (Li et al., 2015). Then, the genetic editing started with cotransformation of donor DNA and gRNA plasmid into preceding cells. Comparing to the previously established system, this optimized system has a higher gene editing efficiency and less operating time, almost 100% for codon replacements and knockout genes within 2 days. It was noteworthy that using a double-strand DNA as a donor template has a better performance than a single-strand DNA in gene deletions. Subsequently, this optimized system was employed to strengthen the MEP pathway by substituting the promoters and ribosome binding sites, inserting a heterologous β-carotene biosynthetic pathway and optimizing the central carbon metabolism (Li et al., 2015). Finally, the best producer yielded 2.0 g/L β-carotene in fed-batch fermentation. This extensive work can hardly be completed without employing CRISPR-based tools, revealing their great potential for efficient and diverse manipulation of genomic DNA. The Cas9-recombineering method was further exploited with the development of the CRISPR-enabled trackable genome engineering (CREATE) tool (Garst et al., 2017). Application of this tool in *E. coli* allowed for simultaneous transformation of multiple libraries of plasmid-borne recombined templates (Garst et al., 2017). The CREATE strategy was employed to construct genome libraries of isopropanol pathway by introducing multiple ribosome binding site variations in *E. coli*, leading to the construction and testing of ~1,000 strains in a few days. The best performer reached a titer of 7.1 g/L isopropanol within 24 h (Liang et al., 2017).

Besides the versatile applications in *E. coli*, the CRISPR-based tools also had satisfactory performances in other bacteria (Table 1). For instance, genetic engineering technologies for solventogenic *Clostridium* were still immature due to low transformation efficiency, inadequate endogenous homologous recombination, and poorly understood physiology and metabolism (Papoutsakis, 2008; Pyne et al., 2014; Bruder et al., 2016). Recently, CRISPR/Cas9 for *Clostridium saccharoperbutylacetonicum* N1-4, a hyper-butanol-producing strain, was developed. The genome engineering efficiency was improved from 20 to 75% by selecting optimized promoter P_J23119_ from *E. coli* for gRNA expression. After deleting two essential genes of phosphotransacetylase (*PTA*) and butyrate kinase (*BUK*) for acetate and butyrate production, the butanol production reached 19.0 g/L, which is one of the highest levels ever reported from batch fermentations (Wang et al., 2017). This Cas9-based editing tool could be easily adapted for use in closely related microorganisms, paving the way for elucidating the mechanism of solvent production and constructing robust strains with desirable butanol-producing features. In addition, the Cas9-based editing tools have also been successfully employed for the production of bulk chemicals, such as succinate in *Synechococcus elongatus* (Li et al., 2016), isopropanol-butanol-ethanol in *Clostridium acetobutylicum* (Wasels et al., 2017), and γ-amino-butyric acid (GABA) in *Corynebacterium glutamicum* (Cho et al., 2017). The wide application of the CRISPR/Cas9 system in a variety of bacteria genera demonstrates that it plays a critical role in the prosperous development of bioindustrial.

**Table 1:** Applications of the CRISPR/Cas9 system in bacteria.

**Species**	**Cassettes for CRISPR/Cas9**		**Editing efficiency**	**Advances in genetic modification using CRISPR/Cas9**	**Strategies for improving efficiency**	**References**
	**Cas9 protein**	**gRNA**				
*Bacillus subtilis*	*lac*A5′-Cas9-tracRNA-*lac*A3′	*thr*C5′-P_xylA.*SphI*+1_-gRNA-*thr*C3′	100and 85% for single, double gene mutations, and 69% for chromosomal insertion of a 2.9 kb hyaluronic acid (HA) biosynthetic operon	Multiplex knockout	Choose optimal homology lengths (1,000 bp) for editing template, optimize PAM site	Westbrook et al., 2016
	P*grac*-*Sp*Cas9	Para-sgRNA-donor DNA	Point mutation (68%), single-gene deletion in spo0A (100%), and gene insertion (97%)	Traceless, high efficient	Incubation for longer periods to generate iterative DSB	So et al., 2017
*Clostridium autoethanogenum*	Cas9 was introduced into plasmid pLZtet3no and pIPL12	sgRNA was introduced into plasmid pMTL83157	Over 50% for gene deletion	Construct a small library of tetracycline-inducible promoters for efficient gene deletion	Construct variants of inducible promoter to control the expression of Cas9. Cas9 protein was codon adapted to *C. autoethanogenum*	Nagaraju et al., 2016
*Clostridium cellulolyticum*	Optimized *Sp*Cas9 was introduced into plasmid pLyc017	gRNA was introduced into plasmid pCR8/GW/TOPO TA	High editing efficiency (>95%)	High editing efficiency even using short homologous arms (0.2 kb), deliver foreign genes into the genome in a single step without a marker	Generate single-nick triggered homologous recombination and choose optimal homology lengths for editing template	Xu T. et al., 2015
*Corynebacterium glutamicum*	P_tac_-SD-*Sp*Cas9 was introduced into plasmid pXMJ19	P_trc_-sgRNA was introduced into plasmid pEC-XK99E	Deletion efficiencies were almost 100% for *porB, mepA, clpX*, and Ncgl0911 genes	High editing efficiency even using short homologous arms (0.3 kb)	Choose strong promoters for the expression of Cas9 and sgRNA	Liu J. et al., 2017
*Clostridium ljungdahlii*	P*_*thl*_*-*Sp*Cas9	P_araE_-sgRNA	Deletion efficiencies were 100%, >75%, 100%, and >50% for *pta, adhE1, ctf*, and *pyrE*	More rapid, no added antibiotic resistance gene, scarless, and minimal polar effects	Choose strong promoters for the expression of Cas9 and sgRNA	Park et al., 2019
*Clostridium pasteurianum*	P*_*thl*_*-*Sp*Cas9	P_sRNA_-gRNA	Deletion efficiencies were 100% for *cpaAIR*	High efficient	Inducible expression of cas9 was recommended to mitigate toxicity for high editing efficiency	Pyne et al., 2016
*Lactobacillus reuteri*	tracrRNA, cas9, and CRISPR arrays derived from pCAS9 were introduced into plasmid pNZ9530		100% for genes mutations	High efficient	Employ oligonucleotide-mediated recombineering (RecT)	Jee-Hwan and Jan-Peter, 2014
*Streptomyces albus*	P*_*rpsLp*_*-Cas9-T_fd_	P*_*gapdhp*_*-gRNA-T*_*fd*_*	Multiplex gene deletions with editing efficiency ranging from 70 to 100%	Reduce the time and labor needed to perform precise genome manipulation	Choose strong promoters for the expression of Cas9 and gRNA; Cas9 gene was optimized to favor the *Streptomyces* codon bias	Wang et al., 2016
*Streptomyces coelicolor*						Wang et al., 2016
*Streptomyces lividans*						Wang et al., 2016
*Streptomyces viridochromogenes*						Wang et al., 2016

### The Application of the CRISPR/Cas9 System in Yeasts

Yeasts play critical roles in industrial biology, as a wide range of products can be produced by yeasts, including biopharmaceuticals, biocatalysts, food additives, fine chemicals, and renewable biofuels (Raschmanova et al., 2018). Due to their robust physiology, yeasts could be cultivated in harsh growth conditions, such as low pH and elevated temperatures. Furthermore, yeasts can be introduced in complex eukaryotic post-translational modification systems, which are absent in bacterial hosts and often vital for biopharmaceuticals production (Thomas et al., 2013). Until now, a series of genetic engineering tools based on CRISPR/Cas9 had been exploited in yeast to improve the efficiency of genetic modification (Mitsui et al., 2019).

For decades*, S. cerevisiae* has been a well-known model organism in research and application areas (Jakočiunas et al., 2016). To verify the efficiency of the CRISPR/Cas9 system in *S. cerevisiae*, the endogenous genomic negative selectable marker *CAN1* (encoding arginine permease) can be chosen as a target gene (Dicarlo et al., 2013). To further improve the gene knockout efficiency, 90-bp double-strand oligonucleotides (dsOligo) including homologous arms to the target site and an internal stop codon as the HR template were designed. The recombined frequency of mutations selected from the medium containing canavanine (a toxic arginine analog that can kill cells containing functional *CAN1* gene) was almost 100%, providing foundations for simple and powerful genome engineering tools in yeasts. This system can be used to knock out *LEU2, TRP1, URA3*, and *HIS3* in polyploid industrial yeast *S. cerevisiae* ATCC 4124 with efficiency of up to 60%, and a quadruple-deficient strain (Δ*ura3*, Δ*trp1*, Δ*leu2*, Δ*his3*) was successfully constructed (Zhang et al., 2014). In order to further improve the genetic editing efficiency of the CRISPR/Cas9 system, the USER cloning technology was accordingly employed to assemble multiple sgRNAs in one plasmid for efficient gene disruption and promoter engineering of one to five target loci in one step (Jakočiunas et al., 2015a). This one-step maker-free genome editing approach achieved high efficiencies of 50–100% from single to quintuple edits. However, the low efficiency of cotransformation of gRNA plasmids and corresponding HR donors hindered large-scale genome engineering applications (Lian et al., 2018). To solve this problem, a homology-integrated CRISPR (HI-CRISPR) system by fusing a 100-bp HR template to the 5′ end of the crRNA sequences was constructed, leading to 87% efficiency of multiplex knockout of CAN1, ADE2, and LYP1 (Bao et al., 2015). This HI-CRISPR system further improved the efficiency of multiplex genome editing and genome-scale engineering.

In addition to gene knockout, the CRISPR/Cas9 system can also mediate gene insertion using the homologous arm of donor DNA to the target gene. To better control cellular levels of correctly folding sgRNA, HDV ribozyme was fused to the 5′ end of sgRNA to protect the 5′ end of the sgRNA from 5′ exonucleases. This HDV-gRNA expression strategy significantly increased the efficiency of multiplex genome editing in diploid yeast strains, in which the heterologous cellodextrin transporter (*cdt-1*) and endogenous β-glucosidase (*gh1-1*) were inserted. As a result, the efficiency of utilizing cellobiose was increased by 10 times through site-directed mutagenesis of *cdt-1* and *gh1-1* genes via the multiplexed CRISPR/Cas9 system (Ryan et al., 2014). Meanwhile, Ronda et al. cotransformed one episomal vector expressing three gRNAs with three donor DNAs containing β-carotene synthesis genes of *BTS1, crtYB*, and *crtI* into *S. cerevisiae*, enabling it to synthesize β-carotene (Ronda et al., 2015). Similarly, three exogenous genes (*XYL1, XYL2*, and *XYL3*) encoding for xylose reductase, xylitol dehydrogenase, and xylulokinase from *Scheffersomyces stipites* were integrated into the loci of *PHO13* and *ALD6* in *S. cerevisiae* by employing the CRISPR/Cas9 system (Tsai et al., 2015). The refactored strains achieved the ability of utilizing xylose and could be readily used for large-scale fermentations, as no antibiotic-resistant markers were adopted. In addition, a more versatile genome engineering tool, Cas9-facilitated multiloci integration of *in vivo* assembled DNA parts (CasEMBLR), was constructed by combining DNA assembly, HR of DSBs using donor DNAs, and multiplex gRNA expression cassettes (Jakočiunas et al., 2015b). As a proof of concept, this CasEMBLR was employed to assemble and integrate the gene expression cassettes (upstream homology arm, promoter, structural gene, terminator, and downstream homology arm) of *CrtYB, CrtI*, and *CrtE* into the loci of *ADE2, HIS3*, and *URA3*, with a marker-free engineering efficiency of 31%.

CRISPR/Cas9 was also implemented in many other industrial yeasts (Table 2). For example, to construct more suitable promoters for sgRNA in *Schizosaccharomyces pombe*, Jacobs et al. constructed an expression cassette by adding the leader RNA from *rrk1* gene between the promoter and sgRNA sequence, and fused the HH ribozymes with 3′ end of the mature sgRNA to achieve correct 3′ sgRNA processing. This system achieved a high efficiency of 98% when a donor template was cotransformed. To further simplify the operation of the CRISPR/Cas9 system in *S. pombe*, Zhang et al. developed a cloning-free procedure including a gapped Cas9-encoding plasmid and a PCR-amplified sgRNA insert. *Ura4* and *rrk1* promoter-leader was just between the gap of Cas9-encoding plasmid. The PCR-amplified sgRNA insert was consisted of sgRNA target sequence and scaffold, and flanked with the homologous arms of *ura4* and promoter-leader. Accordingly, a circular plasmid including Cas9 and sgRNA could generate two gap-repairing fragments. This cloning-free procedure could change the sgRNA target sequence only using an 83-bp sgRNA premier instead of cloning the whole sgRNA plasmid.

**Table 2:** Applications of CRISPR/Cas9 system in yeast.

**Species**	**Cassettes for CRISPR/Cas9**		**Editing efficiency**	**Advances in genetic modification using CRISPR/Cas9**	**Strategies for improving efficiency**	**References**
	**Cas9 protein**	**gRNA**				
*Kluyveromyces lactis*	P*_*FBA*1_*-*Sc*CAS9-*CYC1TT*	P*_*SNR*52_*-gRNA-*SUP4TT*	Multiple-gene cassette insertion into multiple-gene loci: 2.1%	Multiplex knock-in	Delete *KU80* gene to increase the frequency of HR	Horwitz et al., 2015
*Komagataella pastoris*	P*_*HTA*1_*-*Hs*CAS9-*DAS1TT*	P*_*HTB*1_*-HH ribozyme-gRNA-HDV ribozyme-*AOX1TT*	87–94% for single-gene disruption and 69% for double-gene disruptions	Multiplex knockout	Choose optimal promoters for the expression of Cas9 and gRNA	Walter et al., 2016
*Kluyveromyces marxianus*	P_Tef1p_-Cas9-*CYC1TT*	P_RPR1−tRNAGly_-sgRNA	66% for single-gene disruption	High editing efficiency	Choose optimal promoters for the expression of sgRNA	Löbs et al., 2017
*Ogataea polymorpha*	P*_*TDH*3_*-Cas9	P*_*tRNA*_*-sgRNA	45% for single-gene disruption	High editing efficiency	Modify system using a tRNA-sgRNA fusion gene to increase the mutation efficiency	Numamoto et al., 2017; Wang et al., 2018
*Schizosaccharomyces pombe*	P*_*ADH*1_*-*Hs*CAS9-*CYC1TT*	P*_*RRK*1_*-rrk1 leader-gRNA-HH ribozyme	Single-gene disruption (allele swap): 85%−90%	Highly efficient knockout	Add the leader RNA from *rrk1* gene between the promoter and the gRNA sequence	Jacobs et al., 2014
*Yarrowia lipolytica*	P*_*UAS*1*B*8−*TEF*(136)_*-*Yl*Cas9	${\text{P}}_{SCR1^{\prime }-tRNAGly}$ –gRNA-poly T	Single-gene disruptions (NHEJ/HR): 90–100%/64–88%	Highly efficient knock out	Choose optimal promoters for the expression of sgRNA; delete *KU70* gene to increase the frequency of HR	Schwartz et al., 2015
	P*_*TEFin*_*-HsCAS9-*CYC1TT*	P*_*TEFin*_*-HH ribozyme-gRNA-HDV ribozyme-MIG1TT	Single-gene disruption (NHEJ/HR): 62%−98%/72%; multiple-gene disruptions (NHEJ):19–37%	Highly efficient, scarless, single or multigene editing	Choose strong promoters for the expression of Cas9; delete *KU70* gene to increase the frequency of HR; Cas9 protein was codon adapted to *Y. lipolytica*	Gao et al., 2016

### The Applications of the CRISPR/Cas9 System in Filamentous Fungi

Due to the considerable economic value of their metabolites, filamentous fungi have been applied to produce antibiotics, organic acids, pigments, polyunsaturated fatty acids, and so on (Dufossé et al., 2014; Ji et al., 2014; Xu X. et al., 2015). However, challenges of delivery through fungal cell wall and lack of available promoters and plasmids hindered the development of genetic engineering tools in filamentous fungi. In addition, the low editing efficiency and consequent large amount of labor time impeded the further application of filamentous fungi in the industry. The emerged CRISPR/Cas9 has brought a breakthrough for genetic manipulation in filamentous fungi.

Conventional genetic engineering strategies to improve the efficiency of HR were to delete *KU70* or *KU80* heterodimer (Weld et al., 2006). However, if *KU70* or *KU80* is interrupted, the filamentous fungi will become more sensitive to growth environments with specific requirements for some chemicals, such as phleomycin, bleomycin, and methyl/ethyl methanesulfonate (Liu et al., 2015). To overcome this obstacle, the CRISPR/Cas9 system in the filamentous fungus of *Trichoderma reesei* using specific optimized codon and *in vitro* RNA transcription was established (Hao and Su, 2019). The highest frequency of single HR in *T. reesei* using a pair of ≥600-bp homology arms was almost 100%. Subsequently, a microhomology-mediated end-joining system based on CRISPR/Cas9 in *Aspergillus fumigatus* was also established by flanking the sgRNA with HH and HDV. This system achieved accurate target gene editing with a high efficiency of 95–100% via very short (~35-bp) homology arms, indicating that it can function as a powerful and versatile genome editing tool in *A. fumigatus* (Zhang et al., 2016). In addition to the successful application in different species of *Aspergillus*, this evolved system has also been employed in several different filamentous fungal species, broadening the application of the CRISPR/Cas9 system (Hao and Xia, 2015; Weber et al., 2016; Weyda et al., 2017).

Taken together, the CRISPR/Cas9-mediated gene editing technology not only efficiently edited individual target gene but also showed satisfactory performances in multigene editing, which greatly promoted the development of genetic manipulations in filamentous fungi.

## Combination of Cas9-Mediated Transcriptional Regulation With Synthetic Biology

In addition to the overexpression or deletion of genes, which occur in desired metabolite pathways, regulation of gene expression at the transcription levels is also very important to obtain high yields of metabolites (Mougiakos et al., 2018). Conventional methods to regulate gene expression were to use different promoters with desired strength or RNA interference (Crook et al., 2014). However, with the increased numbers of target genes, the task of testing promoters with suitable strength was time-consuming and labor-intensive. In addition, in order to achieve transcriptional control in terms of level and timing, a complex and sophisticated genetic circuit including activating and repressive transcription factors is indispensable. According to the unique features of the CRISPR/Cas9 system, Jinek et al. constructed a catalytically deactivated Cas9 (dCas9) by introducing inactivating mutations into two nuclease domains of the Cas9 endonuclease, one in the HNH nuclease domain (H840A) and the other in the RuvC-like (D10A) domain (Jinek et al., 2012). This dCas9 protein lost the activity of DNA cleavage but retained the ability to specifically bind to target DNA sequences complementary to the sgRNA. Soon after, this CRISPR/dCas9 system was employed to synthesize a transcriptional repressor. Qi et al. showed that RNA guiding of dCas9 to target genes or promoters would block RNA polymerase binding and genetic transcription, leading to the repression of gene expression (Qi et al., 2013). This CRISPR interference (CRISPRi) system subsequently was used to create efficient, programmable, and genome-wide scale transcriptional regulators (Gilbert et al., 2013, 2014; Silvana et al., 2015). Similarly, CRISPR activation (CRISPRa) was constructed through the fusion of dCas9 to transcriptional activators or activation domains, allowing for transcriptional upregulation of select genes (David et al., 2013). To investigate whether the system could activate the transcription of reporter gene, dCas9 with an omega (ω) protein was fused, which could improve RNA polymerase activity and activate the transcription in *E. coli*. As a result, the transcription level of the reporter gene was increased by 2.8 times (David et al., 2013). In order to broaden the application of CRISPRa, several transcription activator domains have been excavated, such as VP64 and p65AD (Farzadfard et al., 2013; Silvana et al., 2015). Therefore, CRISPRi/a was allowed for facile transcriptional modification of gene networks and satisfied as an important tool to control enzyme expression levels in endogenous or synthetic pathways (Donohoue et al., 2017).

The core concept of synthetic biology is to transform existing natural systems and construct gene circuits by building and integrating standardized components and modules (Dai et al., 2018). However, an ultimate challenge in the construction of gene circuits was lack of effective, programmable, secure, and sequence-specific gene editing tools. The CRISPRi/a system was poised to solve this problem owning to its programmable targeting, efficacy as activator or repressor, high specificity, and rapid and tight binding kinetics (Sternberg et al., 2014; Xu and Qi, 2019). The CRISPR/Cas9 transcriptional regulator (CRISPR-TF) has been developed to become an important component of scalable device libraries, which were essential in creating complex genetic circuits. Nissim et al. constructed a multi-RNA-mediated gene network regulatory toolkit including RNA-triple-helix structures, introns, microRNAs, ribozymes, Cas9-based CRISPR-TFs, and Cas6/Csy4-based RNA processing to perform tunable synthetic circuits (Nissim et al., 2014). Nielsen et al. also constructed multiple transcriptional logic gates by employing CRISPR/dCas9 and linked them to perform logical computations in living cells (Nielsen and Voigt, 2014). In specific applications, researchers constructed a set of NOT gates by designing five synthetic σ70 promoters in *E. coli*. These promoters were inhibited by the corresponding sgRNAs, and interrelationships between various components did not exhibit crosstalk. Furthermore, they used these NOT gates to build larger lines, including the Boolean-complete NOR gate and three gates, which were consisted of four-layered sgRNAs. The previously designed gene synthetic lines were ligated into the native regulatory network using an export sgRNA capable of targeting the transcriptional regulator (MalT) in *E. coli*. By using these methods, the output of synthetic lines can be converted into switches of various cell phenotypes, such as sugar utilization, chemotaxis, and phage resistance.

Comparing with traditional methods of regulating expression, these complex gene circuits based on CRISPRi/a could carry out more precise regulation in terms of levels and timing. In addition, a cascade of responses in these circuits could be triggered by only one signal, which is more advanced than complex promoter engineering. Implementation of CRISPRi/a to design precise gene circuits in biosynthesis pathways will facilitate an unprecedented level of control of metabolic flux and promote the rapid development of the bioindustry.

## Conclusion and Future Directions

In the past, non-model microbes have been greatly limited in product diversity and yield due to the lack of efficient genetic engineering tools. These problems could be solved under the wide application of the CRISPR/Cas9 system, which will bring a bright future in the industrial biotechnology. Comparing to the conventional marker-based genome editing tools, the CRISPR/Cas9 system enabled fast strain engineering of prototrophic wild and industrial strains, allowing for multiple genome editing simultaneously with marker cassette integration (Stovicek et al., 2017). The high-throughput screening of industrial strains was another bottleneck in industrial biology (Donohoue et al., 2017). Recently, droplet-based microfluidics technology attracted great attention in terms of strain isolation and characterization due to its high-throughput screening efficiency. The combination of the CRISPR/Cas9 system and droplet-based microfluidics technologies maybe bring new breakthroughs in industrial biology in the future.

Although CRISPR/Cas9-meditated genome editing techniques were increasingly used in biotechnology, some disadvantages still existed (Zhang et al., 2019). First, the off-target effect is still a main issue needed to be solved. The designed sgRNAs may form mismatches with non-target DNA sequences, resulting in unexpected gene mutations. These unexpected gene mutations may cause genomic instability and disrupt the function of other normal genes. Although a variety of methods have optimized off-target effects, further improvement is still needed. Hence, conformational changes associated with sgRNA and DNA recognition need to be further explored to obtain higher genome editing accuracy (Mir et al., 2017). In addition, time-dependent control of Cas9 protein activity and the ratio of Cas9 to sgRNA also require further investigation. High expression of Cas9 protein, whether in gene knockout or gene insertion, will affect the growth and recovery rate of host bacteria after transformation, and cause fatal damage to cells in most cases. The existing methods to increase the HR frequency are not suitable to many non-conventional industrial microorganisms. Hence, exploitation of novel approaches to improve homologous recombination efficiency in these strains is critical for precise and efficient CRISPR/Cas9-mediated genetic engineering. Besides, the intellectual property and scientific ethics issues also hindered the wide application of CRISPR/Cas9. The patent dispute has come to an end in the United States, but the war is continuing in Europe and other countries, delaying its adaptation for industrial biotechnology and pharmaceutical applications. Just like the first test-tube baby that appeared in 1978, employing CRISPR/Cas9 for gene therapy also caused some controversies. Therefore, if the laws in the application of the CRISPR/Cas system were completed, it would promote rapid development in the fields of medicine, genomics, agriculture, and so on.

## Author Contributions

SZ conceived, designed, and drafted the paper. SZ and FG wrote the part of the application of CRISPR-Cas9 in bacteria. WY and ZD wrote the part of the application of CRISPR-Cas9 system in Yeast. JZ and WD wrote the part of the applications of CRISPR-Cas9 system in filamentous fungi. MJ, WZ, and FX wrote the part of Cas9-mediated transcriptional regulation and critically revised the manuscript. All authors read and approved the final manuscript.

### Conflict of Interest

The authors declare that the research was conducted in the absence of any commercial or financial relationships that could be construed as a potential conflict of interest.
